# The transcription map of HPV11 in U2OS cells adequately reflects the initial and stable replication phases of the viral genome

**DOI:** 10.1186/s12985-015-0292-6

**Published:** 2015-04-14

**Authors:** Helen Isok-Paas, Andres Männik, Ene Ustav, Mart Ustav

**Affiliations:** Institute of Technology, University of Tartu, Tartu, Estonia; Icosagen Cell Factory OÜ, Tartu, Estonia; Estonian Biocentre, Tartu, Estonia; Estonian Academy of Sciences, Tallinn, Estonia

**Keywords:** Transcriptome, Genome replication, HPV11, HPV, Papillomavirus

## Abstract

**Background:**

Although prophylactic vaccines have been developed against HPV6, HPV11, HPV16 and HPV18 there is the clear unmet medical need in order to justify the development of drugs targeting human papillomavirus replication. The native host cells of HPVs are human primary keratinocytes which can be cultivated in raft cultures. However, this method is difficult to use in high-throughput screening assays and the need for a cost-effective cellular system for screening potential anti-HPV drug candidates during all stages of HPV genome replication remains.

**Methods:**

U2OS cells were transfected with HPV11 wt or E8- minicircle genomes and their gene expression was studied via 3′ RACE, 5′ RACE or via real time PCR methods. The DNA replication of these genomes was detected by Southern blot methods.

**Results:**

The analysis of HPV11 transcripts in U2OS cells showed that the patterns of promoter use, splice sites and polyadenylation cleavage sites are identical to those previously characterized in human HPV-related lesions, human squamous carcinoma cell lines (e.g., SSC-4) and laryngeal papillomas. Transcriptional initiation from the three previously described HPV11 promoters in the E6 and E7 ORFs (P90, P264, and P674-714) were functional, and these promoters were used together with two promoter regions in the E1 ORF (P1092 and P1372). Mutating the E8 ORF ATG start codon to ACG eliminated the translation of fusion proteins from the E8 ORF coupled to E1 and E2 proteins C-terminal sequences, leading to the de-repression of gene expression (particularly from the P1092 promoter) and to the activation of genome replication. These data suggested that the expression of the functional E8^E2 protein is used to control viral gene expression and copy number of the HPV11 genome. The analysis of HPV11 E1 expression plasmids showed that the E6/E7 region, together with the E1 coding region, is crucial for the production of functionally active E1 protein.

**Conclusions:**

The data presented in this paper suggest that in human osteosarcoma cell line U2OS the gene expression pattern of the HPV11 truly reflect the expression profile of the replicating HPV genome and therefore this cellular system is suitable for drug development program targeting HPV replication.

## Background

Papillomaviruses are small double-stranded DNA viruses that are classified into 16 genera according to their genotypes [1,2]. The best-studied PVs belong to the group of alpha-subtype viruses (α-PVs) that infect the oral and anogenital mucosa of humans and that can cause condylomas, genital warts (low-risk α-HPVs, such as HPV6 and 11) or anogenital cancers (high-risk α-HPVs, such as HPV16, 18 and 31). The organization of the HPV genome, which is conserved among PVs, contains early (E) and late (L) coding regions and a non-coding long control region (LCR) also known as the upstream regulatory region (URR). The early region genes are expressed at different levels throughout the whole infection period and are responsible for virus DNA replication (E1 and E2), transcriptional regulation (E2) and cellular regulation/transformation (E6, E7, E4, and E5). The late genes, which encode the viral capsid proteins L1 and L2, are expressed in terminally differentiated keratinocytes during the productive phase of infection. The non-coding upstream regulatory region contains *cis*-elements for cellular factors, as well as for viral DNA replication, transcription and genome maintenance. The DNA replication cycle of PVs can be divided into 3 phases: initial genome amplification, during which the copy number increases rapidly in infected cultures to up to 50-100 copies per cell; the latent replication phase, during which the viral genome is replicated in synchrony with the cellular DNA; and the vegetative phase, which includes the second genome amplification, viral capsid proteins expression and virion assembly.

Two vaccines against α-papillomaviruses have been developed and marketed as Cervarix (GlaxoSmithKline, UK) and Gardasil (Merck Research Laboratories, US). For review see Malik et al. [3]. These vaccines induce protective neutralizing antibodies against L1 protein epitopes and target the high-risk viruses HPV16 and HPV18, which are the etiological agents responsible for the induction of cervical carcinoma. Gardasil also provides protection against genital warts and cancers of the anus, vagina and vulva by targeting the low-risk viruses HPV6B and HPV11. These vaccines are highly effective in raising specific immune responses and provide protection against HPV6B, HPV11, HPV16 and HPV18 viruses when vaccinations are performed in young girls before sexual exposure [4,5]. The factors preventing the wide and effective use of the current HPV vaccines include their cost and their partial protection against many other papillomavirus types. Although, cross-protection for closely related HPV types like HPV31 and 45 has been observed [6] there are at least 13 HPV types which are potentially oncogenic [7]. Also, current vaccines do not generate therapeutic effect against pre-existing HPV infection. Therefore, a real unmet medical need for drugs with a wider spectrum of specificity against most papillomaviruses exists.

One of the more specific targets for therapeutic intervention against HPV infection could be the replication machinery of HPVs. The wide variety of papillomaviruses can be specifically targeted for drug intervention by focusing on the expression of the viral E1 and E2 proteins and their interaction with cellular proteins and specific features of the replication machinery. Such drugs may show wide specificity for HPVs because of the great similarities in the interactions of the E1 and E2 proteins of different HPV subtypes with their specific cellular counterparts and DNA sequences. Thus, drugs developed against specific HPV replication stages may have a specific effect on a wide variety of HPVs. To screen for such HPV replication inhibitors, a cell-based assay must be developed in which HPV genome replication can be effectively quantitated in a high-throughput screening (HTS) format.

The initial phases of PV infection until the generation of viral particles can be studied in raft cultures using transfected PV genomes in human primary keratinocytes or squamous carcinoma cell lines (e.g.*,* SCC-4) [8]. Alternatively, naturally derived cell lines like W12 (HPV16) or CIN612 (HPV31) already harboring replicating HPV DNA episomes allow the latent and vegetative phases of the PV life cycle to be studied [9-11]. Although investigating the molecular mechanisms of HPV replication in raft cultures is important for a complete understanding of viral genome replication in differentiating cells of specific tissues, this method is difficult to use for screening potential drug candidates in HTS assays. This issue also applies to the use of primary keratinocyte cultures for HPV replication because of the need for donors of primary cells, in addition to issues concerning the genetic uniformity of the assay. Alternatively, NIKS cells which are non-HPV-containing immortalized keratinocytes could be used to develop an HTS assay, although the robustness of this methodology must be improved for the effective use of this system [11,12].

Previously, we have successfully employed the human osteosarcoma U2OS cell line to analyze genome replication and gene expression in α- and β-HPVs [13-16]. The initial amplification and latent phases of stable PV replication can be monitored effectively and the subcloning of stable HPV cell lines can be performed in this cell line. Additionally, cloned HPV cell lines can be cultured under dense conditions, thereby triggering the second amplification phase in the case of α-HPVs, which is reminiscent of the vegetative amplification that occurs during the HPV life cycle before late genes expression [13]. However, virus particle assembly has not been detected in these cells because sufficient expression of the capsid proteins L1 and L2 cannot be induced [15,17]. Transcription maps of HPV18 and HPV5 have been compiled in the U2OS cell line [15,17] and compared with previous studies [18,19]. This comparison concluded that transcription maps of these viruses in U2OS cells and in the keratinocytes *in vivo* are very similar, if not identical. Therefore the construction of a high-throughput screening system for inhibitors of the gene transcription and genome replication processes of these viruses in U2OS cells could be possible [15,17,18].

The primary aims of this work were to elucidate the molecular mechanisms of viral gene expression and genome replication further and to confirm whether U2OS cells can be used as a convenient system for molecular studies of HPV11 and as a platform for screening antiviral compounds. We found that the gene expression profile of the HPV11 genome in U2OS cells is very similar to the previously described gene expression in keratinocytes [20-27]. Thus, our data suggest that the HPV11 transcription map obtained herein reflects the situation in vivo and confirm that a U2OS-based system is potentially suitable for screening anti-HPV11 drugs that suppress viral DNA replication.

## Results

### Replication of HPV11 genomic DNA in the human osteosarcoma U2OS cell line

**Figure 1:**
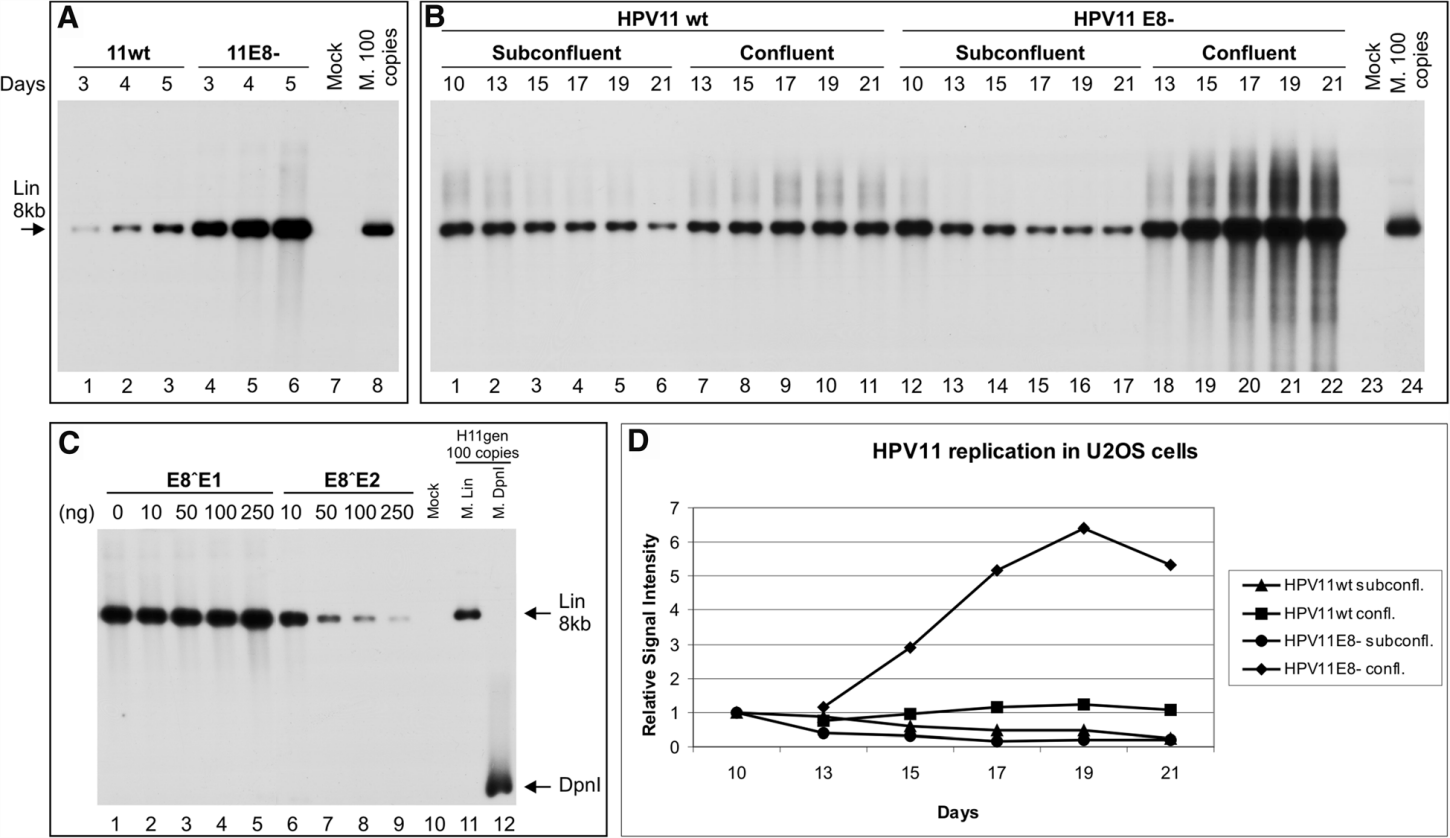
Transient, stable and amplificational replication of the HPV11wt and HPV11E8- genomes in U2OS cells. The mock-transfected cells were used as a negative control (A, lane 7, B, lane 23 and C, lane 10). The linearized HPV11 genome of 100 copies (A, lane 8, B, lane 24 and C, lane 11) and DpnI fragments (C, lane 12) was used as size markers, also indicated with arrows. **(A)** U2OS cells were transfected with 500 ng of the HPV11wt (lanes 1-3) or E8- (lanes 4-6) genome. Extrachromosomal DNA was extracted via Hirt lysis at 3, 4 and 5 days post-transfection, digested with HindIII and with DpnI. The replication signal was detected via the Southern blot method with a radiolabelled HPV11 genome probe. **(B)** U2OS cells were transfected with 500 ng of the HPV11wt (lanes 1-11) or HPV11E8- (lanes 12-22) genome, together with 2 μg of the linearized pBabe-Neo construct. The transfected cells were selected with the antibiotic G418, and at 10 days post-transfection, the cells were split and cultivated under either subconfluent (lanes 2-6 and 13-17) or confluent conditions (lanes 7-11 and 18-22). Total DNA was extracted at the time points indicated at top of the figure, and 3 μg of each sample was analyzed as indicated in **A**. **(C)** Effect of E8˄E1 and E8˄E2 proteins on viral genome replication. U2OS cells were transfected with 500 ng of the HPV11E8- genome with increasing amounts of either the E8˄E1 or E8˄E2 expression plasmid. Total DNA was extracted at 4 days post-transfection, and 3 μg of each sample was analyzed as indicated in **A**. **(D)** The quantitation of HPV11wt and E8- genome DNA replication signals at different time points at subconfluent and confluent culture conditions. The signals were normalized to 10^th^ day time point. Shown is one of two independent experiments.

The replication initiation of papillomavirus genomes is primarily determined by the viral replication factors of E1 and E2, whose levels and effectiveness of action are modulated by different viral and cellular factors [28,29]. To establish HPV11 replication in U2OS cells, the HPV11wt genome was transfected into U2OS cells, which were then cultivated for up to 6 days, and episomal DNA was extracted at different time points by Hirt lysis. The mutant genome HPV11E8- (in which ATG in the E8 ORF is replaced with ACG at nt 1242 in HPV11 genome) was included in this assay because our previous studies examining the HPV5 and HPV18 subtypes in U2OS confirmed the importance of the E8 ORF-containing the E8^E2 protein in the regulation of HPV gene expression and genome replication [15,17]. DNA was purified from the transfected cells at different time points, treated with DpnI to remove the input genome, digested with the linearizing endonuclease HindIII and analyzed by Southern blot assay using a radiolabelled HPV11 genome probe (Figure 1A). In the first establishment period, the replication of the HPV11wt genome could be readily detected in U2OS cells at 3 days post-transfection, with the signal increasing at later time points (Figure 1A, lanes 1-3). Similar to HPV18 and HPV5, the elimination of expression of the E8-containing fusion proteins considerably increased the amplificational replication of the HPV11 genome (Figure 1A, lanes 4-6).

Next, we focused on the stable replication phase of the genome when the viral genome replication is constant, which is necessary for establishing the latent infection phase, and on the final amplification of the viral genomes, which can be induced by growing the HPV-containing U2OS cells under confluent conditions [13]. Thus, the HPV11wt or HPV11E8- genome was co-transfected into U2OS cells with the linearized pBabe-Neo plasmid. Then, the transfected cells were cultivated for up to 1 week under geneticin (G418) selection to eliminate untransfected cells. After selection was complete, the cells were cultivated at lower concentrations of G418 under two different cell culture conditions. Subconfluent (70-90% confluency) conditions were used to analyze the latent replication of the viral genome, whereas confluent conditions (100% confluency) were used to analyze the final amplification. Total DNA was extracted at the indicated time points, after which the DNA was purified and linearized with HindIII, and equal amounts of the DNA samples were analyzed using the Southern blot method, as described above (Figure 1B). Latent persistence with a decrease of the HPV11wt genome copy number was observed during active passaging of the cells under subconfluent conditions from day 10 to day 21 (Figure 1B, lanes 1-6). Although the viral DNA replication level of the HPV11E8- genome on day 10 was higher than that of the wt genome at the same time point (Figure 1B, compare lanes 1 and 12), we observed a similar tendency of decreasing genome copy numbers (Figure 1B, lanes 13-17). This finding suggests that factors other than the E8 ORF product containing proteins are involved in the control of HPV11 copy numbers maintenance during the latent phase.

We observed HPV11wt genome persistence when the cells were cultivated under confluent conditions (Figure 1B, lanes 7-12, Figure 1D). However, the copy number increase was significant in the case of the E8- genome- up to 5-6 times (Figure 1B lanes 18-22, Figure 1D), providing evidence of the regulatory role of E8 ORF-containing fusion proteins in this phase of replication. Collectively, the data suggest that U2OS cells are capable of establishing episomally replicating copies of the HPV11 genome and that different phases of replication (latent replication and final amplification) can be modelled depending on the cultivation conditions.

### The HPV11 E8^E2 protein has a negative effect on viral DNA replication in U2OS cells

E8**^**E2 transcripts have been identified in many PV subtypes: BPV1 [30], HPV11 [20,21], HPV16 [31-33], HPV18 [18,32], HPV31 [32,34] etc. Previous mapping studies of HPV5 and HPV18 transcripts in U2OS cells revealed the following two mRNAs encoding fusion proteins containing the E8 ORF: E8**^**E1, which contains the E8 ORF, the E1 C-terminus and the E2 ORF; and E8**^**E2, which contains the E8 ORF and the E2 C-terminus. E8**^**E2 fusion protein influences viral transcription and genome copy number control [15,17]. The analysis of viral sequences and published transcript maps (http://pave.niaid.nih.gov/#explore/transcript_maps) revealed that the E8**^**E1 and E8**^**E2 proteins could also be expressed by the HPV11 genome. Thus, we engineered expression vectors for the proteins and determined the effect of their expression on HPV11E8- genome replication to investigate the effects of these hypothetical proteins on the regulation of viral genome replication. The co-transfection of increasing amounts of either the E8**^**E1 or E8**^**E2 expression vector with the HPV11E8- genome revealed that the expression of E8**^**E1 did not affect HPV11E8- genome replication (Figure 1C, lanes 2-5). However, HPV11E8- genome replication was suppressed by the E8**^**E2 protein in a concentration-dependent manner, suggesting that HPV11 genome replication can be greatly modulated by this protein (Figure 1C, lanes 6-9). These data are consistent with previous reports demonstrating that the HPV11 E8^E2 protein acts as an important negative regulator of transient DNA replication and gene expression, as observed in α-papillomaviruses, HPV16 [31,35], HPV18 [13,17] and HPV31 [31,34], as well as in the β-papillomavirus HPV5 [15].

### Mapping of HPV11 polyadenylation cleavage sites (CSs)

**Figure 2:**
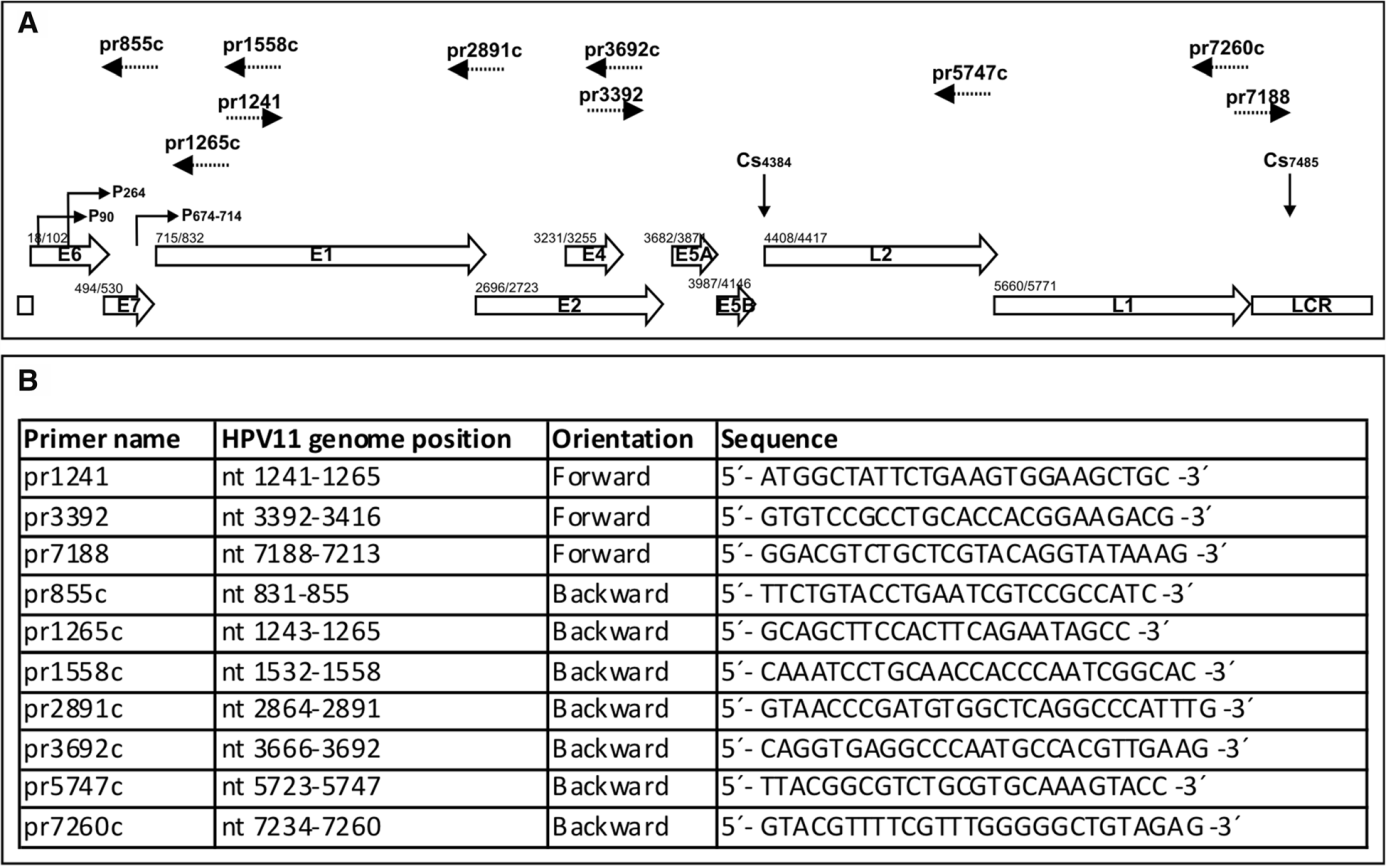
Schematic map of the HPV11 genome and primers used in RACE and RT-PCR analysis. **(A)** Linear depiction of the HPV11 genome, showing the open reading frames (ORFs), long control region (LCR), promoters (P90, P264 and P674-714) and polyadenylation CSs (Cs4384 and Cs7485). The number before each ORF indicates the first nucleotide of the start codon. The primers used in transcriptome analysis are indicated at the top of the figure with dashed arrows. The number given with each primer represents its 5´ end binding position in the HPV11 genome. **(B)** Sequences of primers used for RACE and RT-PCR analysis. The position of each primer in the HPV11 genome and its orientation and sequence are indicated.

Previously, HPV11 gene transcription has been studied in human clinical samples [20,21,24,27] and in a human squamous carcinoma cell line [26] employing various detection mechanisms as PCR [21,23,26], cDNA cloning [27], nuclease mapping [24,25], retrovirus mediated gene transfer [22,23] and RACE analysis [26]. To compare viral transcription in U2OS cells with these previous findings, we decided to map all of the transcripts of the HPV11 genome generated during its replication in these cells. Thus, we extracted polyA^+^ mRNA from U2OS cells transfected with the HPV11wt or HPV11E8- genome and analyzed the samples by 3′ RACE, 5′ RACE, and RT-PCR analyses. The primer sequences used in this study and their positions in the HPV11 genome are shown in Figure 2.

**Figure 3:**
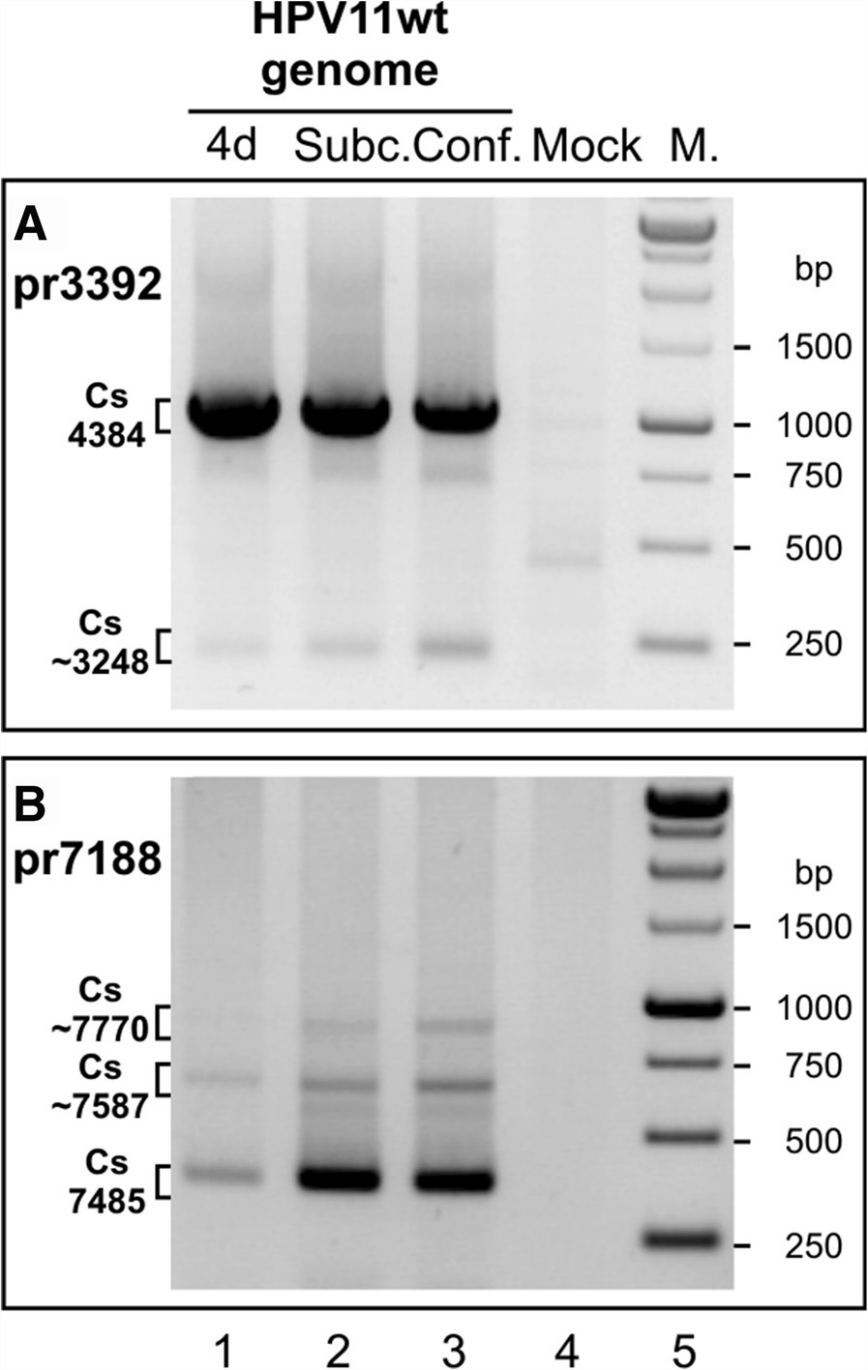
Mapping of polyadenylation CSs in the HPV11 genome in U2OS cells via 3´ RACE. U2OS cells were transfected with the HPV11wt genome (A and B, lane 1) or together with the linearized pBabe-Neo construct (A and B, lanes 2 and 3). Mock transfection was used as a negative control (lane 4). PolyA^+^ mRNA was extracted at 4 or 10 days post-transfection. All distinct products were purified, cloned, sequenced and analyzed. **(A)** 3´ RACE analysis of the HPV11 early region CSs using the HPV11-specific primer pr3392. The indicated 3´ RACE products represent the potential CSs at nt 4384 and nt ~3248. **(B)** 3´ RACE analysis of the HPV11 late region CSs using the HPV11-specific primer pr7188. The indicated 3´ RACE products represent the potential late CSs at nt 7485, at nt ~7770 and at nt ~7587.

The putative HPV11 early polyadenylation CS has been mapped to the downstream region of the viral E5B ORF at nt 4384, and the late CS has been mapped to the downstream region of the L1 ORF at nt 7458 in the HPV11 genome [20,27]. The HPV11 polyadenylation CSs used in HPV11-transfected U2OS cells were mapped via 3´ RACE analysis of polyA^+^ mRNA samples extracted at 4 or 10 days (subconfluent and confluent samples) post-transfection. The HPV11-specific primers pr3392 and pr7188 were used, and the results are shown in Figure 3A and B, respectively. PolyA^+^ mRNA from mock-transfected cells was used as a negative control (Figure 3, lane 4). The sequencing of the clones revealed that the polyadenylation CSs previously described for HPV11 genome transcripts are also used in U2OS cells. We did not observe any major changes when different phases of replication were compared; however, the late polyadenylation CS was used more frequently (Figure 3B).

In addition, we detected three additional CSs at ~3248 (Figure 3A), ~7587 and ~7770 (Figure 3B). These sites were previously unknown, and their functionality remains unclear.

### Mapping of the transcriptional start sites (TSSs) of the HPV11 transcripts in U2OS cells

**Figure 4:**
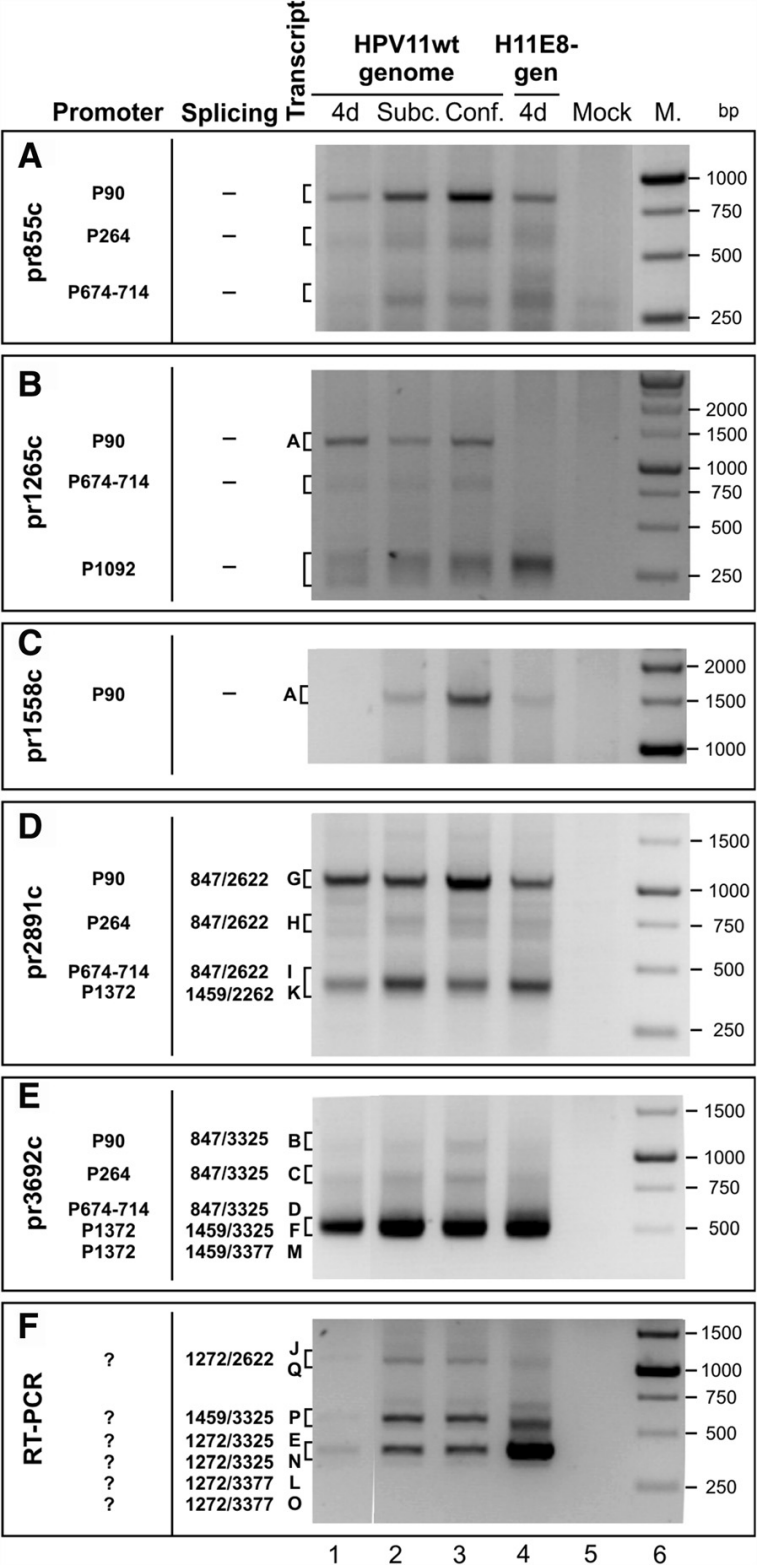
Mapping of HPV11 TSSs and splice junctions in U2OS cells via 5′ RACE. U2OS cells were transfected as follows: 500 ng of the HPV11wt genome (lane 1), 500 ng of the HPV11wt genome together with 2 μg of the linearized pBabe-Neo construct (lanes 2 and 3), 500 ng of the HPV11E8- genome (lane 4), or mock transfection (lane 5). PolyA^+^ mRNA was extracted at 4 and 10 days post-transfection, and 5′ RACE analysis was performed using the HPV11-specific primers pr855c **(A)**, pr1265c **(B)**, pr1558c **(C)**, pr2891c **(D)** and pr3692c **(E)**. RT-PCR analysis was performed using the HPV11-specific primers pr1241 and pr3692c **(F)**. All distinct PCR products (marked with letters from A to Q; the same designations are used in the map represented in Figure 5) were cloned and sequenced for the analysis of TSSs and/or splice junctions.

The 5´ ends of HPV11 transcripts from transfected U2OS cells were mapped via 5´ RACE using the HPV11-specific primers 855c, 1265c and 1558c (Figure 4A-C). Data indicating the HPV11 TSSs in U2OS cells were collected from the independent sequences of 125 5′ RACE clones. In general, the results of this analysis clustered the TSSs into the following 5 distinct promoter regions: P90, P264, P742, P1092 and P1372.

The pr855c primer was designed specifically to obtain information regarding the usage of the first three TSSs in the HPV11wt and E8- genomes. Using this primer, the analysis revealed that the P90 promoter region was the most frequently used promoter region in all of the isolated mRNA samples (Figure 4A).

**Figure 5:**
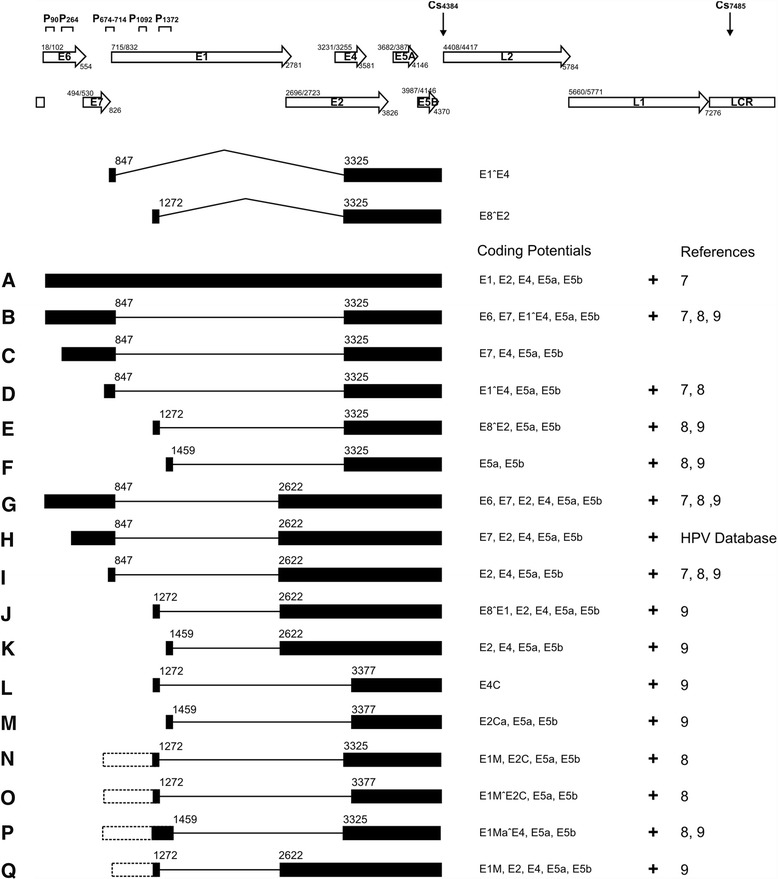
HPV11 transcription map obtained in transiently transfected U2OS cells. The HPV11 ORFs, LCR, promoters (P90, P264, P674-714, P1092, and P1372) and polyadenylation CSs (Cs 4384 and Cs 7485) are shown at the top of the figure. All of the HPV11 transcripts from transiently transfected U2OS cells (indicated with letters from **A**-**Q**, shown at left) are presented, showing the exons (solid boxes), introns (lines) and splicing donor and acceptor sites (nt numbers in HPV11 genome). The inferred 5′ ends of the RT-PCR products **(N-Q)** are indicated with dashed boxes. The coding potential is described to the right of each transcript, and references are provided when similar transcripts were found by others, namely, Chiang et al. [23], Chow et al. [20], and Renaud et al. [26].

The pr1265c primer was specifically designed to detect the TSSs for E8 ORF-containing mRNAs (Figure 4B). Using this primer for 5′ RACE, we detected the following 3 promoter regions: the previously described P90 and P674-714 promoter regions and a potential promoter at P1092, which may be the start site for E8 ORF-containing mRNAs (Figure 5, species E, J and L). Such promoter region was also reported in the studies with HPV16 in human keratinocytes [31]. The transcript starting at P90 may represent the full-length unspliced version of the mRNA, which encodes all of the viral early region proteins, including the viral replication proteins E1 and E2 (Figures 4B and 5, species A). The 5′ RACE product with the TSS at P674-714 might correspond to the unspliced version of the mRNA, which lacks the E6 and E7 ORFs; however, we cannot exclude the possibility of a splicing event at the splice donor site at nt 1459.

The pr1558c primer was designed to specifically detect only the 5′ end of the full-length (unspliced) mRNA. The analysis revealed that the full-length mRNA starts at P90 (Figure 4C). However, we were not able to detect the mRNA with a TSS at P674-714, indicating that transcripts from P674-714 are spliced at nt 1459.

We also detected a putative promoter region at P1372 that has not been previously described as a promoter region (Figure 4D and E). The presence of the promoter region at nt 1374 was suggested by Renaud et al. [26]; however, no additional data were provided. The mRNAs starting at promoter region P1372 potentially encode the viral E2, E4, E5a and E5b mRNAs, depending on the associated splicing events (Figure 5, species F, K, and M). Thus, in U2OS cells, we detected viral transcription from promoters that have also been described in clinical samples. This result strongly support that U2OS cells provide an adequate environment for HPV11 genome replication and gene expression.

In addition, we compared the transcripts obtained during the transient, latent and final amplification phase of replication. We did not observe any major changes in the mRNA pattern. However, regulation of the levels of the mRNA encoding viral replication helicase E1 (Figure 4C, species A, compare lanes 1 and 3) could be detected, which suggests that a second genome amplification occurs due to the intensive transcription of mRNAs encoding the replication proteins.

We also conducted 5′ RACE assays for the HPV11E8- genome to detect differences in transcriptional activity. The most significant differences were detected for transcripts encoding the E1 protein, which were weakly detectable in the HPV11wt genome on day 4 and which were slightly enhanced in the HPV11E8- mutant genome (Figure 4C, compare lanes 1 and 4). RT-PCR analysis with the E8-specific primers pr1241 and pr3962c showed that proteins containing the E8 region negatively regulated their own mRNA transcription (Figure 4F, compare lanes 4 and 1).

### Mapping of HPV11 splice junctions in viral early transcripts

The splice junctions of the HPV11 transcripts found in the polyA^+^ mRNA extracts of transfected U2OS cells were mapped via either 5′ RACE with the HPV11-specific primers pr2891c and pr3692c (Figure 4D and E) or RT-PCR with the primers pr1241 and pr3692c (Figure 4F). In total, 313 mRNA transcripts were cloned, sequenced and analyzed. The splice junction analysis enabled us to identify transcripts that contain splice junctions, embracing all possible combinations of the splice donors at nt 847, nt 1272 and nt 1459 with the splice acceptors at nt 2622, nt 3325 and nt 3377.

The primer pr2891c was designed to detect the 5′ ends of the mRNAs with the splice acceptor site at nt 2622 (Figure 4D). The analysis revealed 4 products, which were designated with letters G, H, I and K. Transcripts G, H and I all exhibited the same splice donor site at nt 847 but different TSSs (P90, P264 and P674-714, respectively). Transcript K was spliced at 1459/2622, and its mRNA started at P1372. All of the products potentially encode the viral E2 protein, product G encodes both the viral E6 and E7 oncoproteins, whereas product H only encodes one oncoprotein, E7 (Figure 5, species G, H, I, K).

The primer pr3692c, which is after the third splice acceptor site at nt 3377, was designed to detect the 5′ ends of mRNAs with the splice acceptor site at nt 3325 or nt 3377 (Figure 4E). Five RACE products were detected when 5′ RACE analysis was conducted with this primer. Three of these products were spliced at 847/3325 (B, C, and D), one at 1459/3325 (F) and one at 1459/3377 (M). The TSSs of these products varied, including P90 (B), P264 (C), P674-714 (D) and P1372 (F and M). Depending on the TSS, the products encode the viral E6 (B and C) and E7 (B) oncoproteins and the E1^E4 (B, C, and D) protein. The most frequently occurred mRNA of these 5 transcripts was species F.

The RT-PCR analysis with pr1241 and pr3692c was designed to detect a rarely used splice donor site at nt 1241. The analysis detected the following 3 rare splicing combinations: 1241/2622, 1241/3325 and 1241/3377 (Figure 4F). The 5′ ends of these species remain unknown; however, some of the mRNAs may be transcribed from the above-described TSS at P1092 and encode the viral E8**^**E2, E8**^**E1 and E4C proteins (Figure 5, species E, J and L, respectively). However, according to previous studies, their TSS may be at the late promoter region P674-714 [23]. If the same TSS is used in the transiently transfected U2OS cell system, then the transcripts encode several truncated forms of E1: E1M, E1MˆE2C and E1MaˆE4 (Figure 5, species N, O, P and Q).

All of the transcripts included in the present study may potentially encode the viral E4 (except for transcripts with an acceptor site at nt 3377), E5a and E5b proteins, at the end of the viral early region (Figure 5).

The results of 5′ RACE analysis using the HPV11-specific primers pr5747c and pr7260c to detect late viral gene products splicing and TSSs included mixed, primarily double-spliced products (data not shown). None of these products were intact to encode the viral structural proteins L1 and L2, indicating that full-length late mRNAs are not expressed and, thus, that viral particles cannot be formed.

### The importance of leader sequences carrying the E6 and E7 ORFs for HPV11 E1 protein expression

HPV E1 protein expression can be regulated by promoter activity, pre-mRNA splicing, mRNA stability, the unusual polycistronic mRNA translation mechanism, nuclear export and import and E1 protein stability. All of these aspects are crucial for effective HPV genome replication during the viral life cycle. In the present study, we focused on identifying the important elements for the transcription of E1 ORF-containing stable mRNAs and then on using this information to achieve functional E1 protein expression. Chow et al. reported that the E1 protein mRNA transcription start site is at the late promoter in the E7 ORF (P674-714) and that the mRNA whose TSS is at nt 90 is believed to either encode the viral E6 protein or be an unprocessed primary transcript of the early region RNAs [20]. HPV11 transcript mapping in the U2OS system revealed that the only mRNA that can encode the E1 protein starts from the early promoter region P90. We have previously demonstrated that the HPV18 E1 protein is translated from spliced polycistronic mRNA, which carries partial spliced sequences of the E6 (generating E6* protein) and E7 ORFs [36,37]. In the present study, we further evaluated the role of the E6/E7-containing leader sequences for HPV11 E1 expression. We designed four different HPV11 E1 expression plasmids. First, we designed an E6/E7 ORF-containing E1 expression plasmid (Leader+, or L+) in which the E6 and E7 translation initiation codons were modified via a single-nucleotide ATG → ACC mutation in both genes. Second, we designed an E1 expression vector lacking the leader sequence of the E6/E7 region (Leader-, or L-) in which the CMV promoter should function as the late viral promoter P674-714, directly in front of the E1 ORF. In designing the third and the fourth E1 expression vectors, we considered the available information for the high-risk viruses HPV16 [38], HPV18 [17,18,36] and HPV31 [39], in which the E6 ORF contains an intron within its sequence. However, a similar intron has not been described in the HPV11 genome. We were interested in determining whether the presence of an intron between the CMV promoter and the E1 region could have any influence on HPV11 E1 protein expression levels. Therefore, we designed E1 expression vectors with a heterologous artificial intron originating from the pRL-TK vector (Promega), which was approximately the same size as the HPV16 E6 intron. We inserted this intron into the E1 (L-) expression vector between the CMV-promoter and the E1 ORF in one or two copies: a single intron-containing vector, designated Int1, and a vector with two introns, designated Int2. The E1 proteins were tagged with an HA-tag for the evaluation of E1 protein expression levels as described previously [36,37].

**Figure 6:**
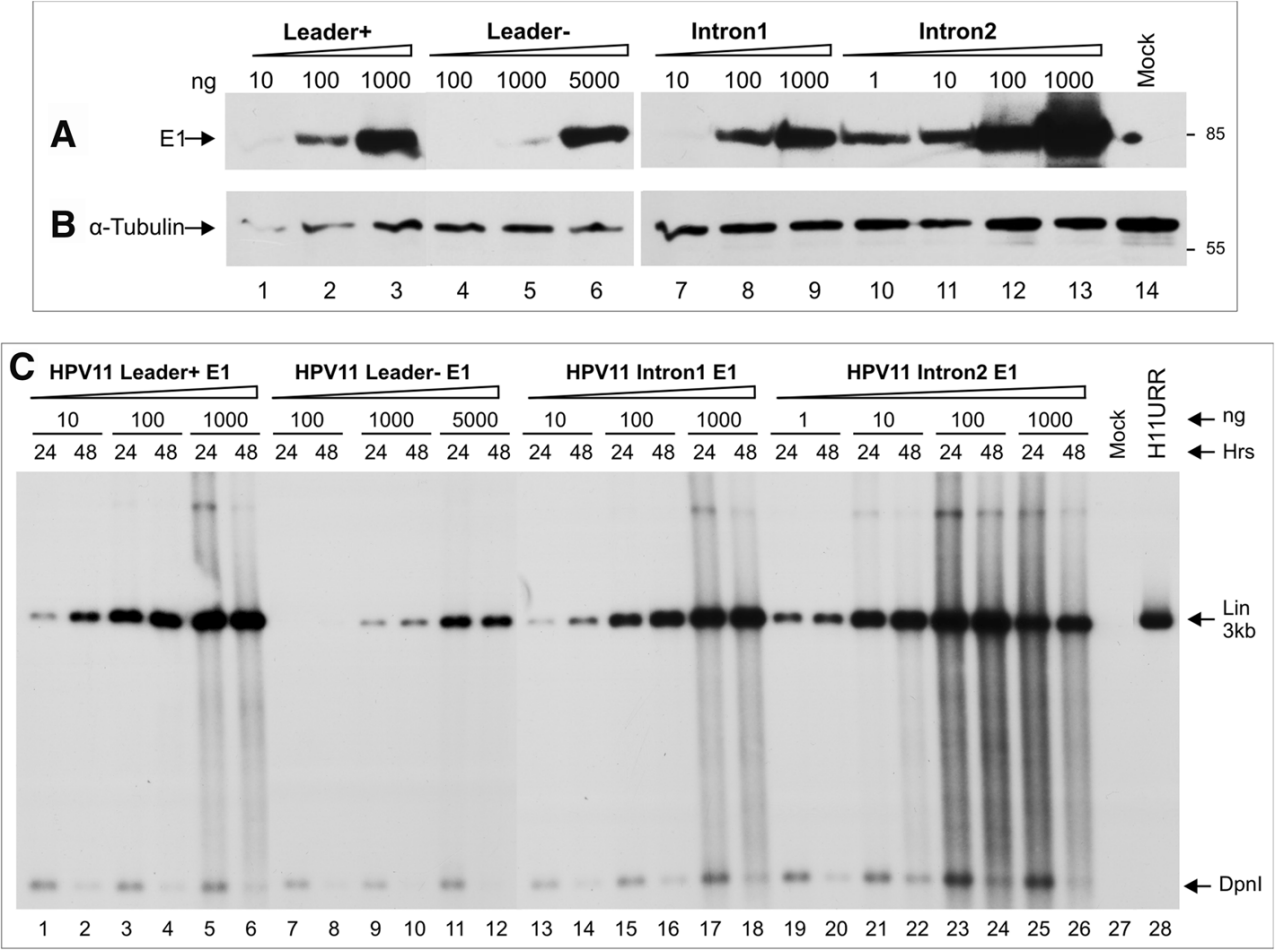
HPV11 E1 expression from E1 plasmids and transient replication of the URR-plasmid in the presence of the E1 and E2 proteins. **(A, B)** Detection of the HA-epitope tagged HPV11 E1 protein from transiently transfected U2OS cells. The cells were transfected with 1-5000 ng of different HPV11 E1 expression plasmids (indicated at the top of the figure). Western blot analysis was performed at the 24 h time point to detect the HPV11 E1 protein **(A)** and the cellular marker α-tubulin **(B)**. **(C)** The HPV11 E1 and E2 proteins initiated DNA replication from the episomal HPV11 URR plasmid in transiently transfected U2OS cells. U2OS cells were co-transfected with 100 ng of the HPV11 URR plasmid, 100 ng of E2 and 1-5000 ng of different E1 expression plasmids. Extrachromosomal DNA was extracted at 24 and 48 h post-transfection via Hirt lysis, and ½ of each sample was analyzed as indicated in Figure 1A. The replication signal was detected with a radiolabelled HPV11 URR specific probe. Mock-transfected U2OS cells were used as a negative control (lane 27), and 200 pg of the linearized HPV11 URR plasmid (lane 28) was used as a size marker. The linear HPV11 URR and DpnI fragments are indicated with arrows.

To test the efficiency of E1 expression from the designed vectors and to identify the effect of the expressed E1 protein on origin-containing plasmids, 100 ng of the HPV11URR plasmid was co-transfected with 100 ng of HPV11E2 and with various amounts of HPV11E1 expression plasmids (10, 100, 1000, or 5000 ng) into U2OS cells. E1 protein expression was evaluated via Western blotting using an HA-tag-specific antibody (Figure 6A). Such tagging of the E1 protein did not interfere with the functions of the protein during replication initiation (data not shown) and enabled us to evaluate E1 protein expression levels. E1 expression levels varied greatly depending on which of the E1 plasmids was used. The E1 protein expressed from the L+ plasmid could be detected when as little as 10 ng of the expression plasmid was transfected (Figure 6A, lane 1), whereas the L- plasmid E1 protein signal was detectable when at least a 100-times higher concentration was used (Figure 6A, lane 5). The plasmid containing one intron expressed E1 at the same level as the L+ plasmid (Figure 6A, compare lanes 1-3 and 7-9), indicating that the natural leader sequence of HPV11 can be substituted with the heterologous intron. The data suggested that sequences preceding the E1 ORF in polycistronic mRNA play an important role in determining E1 protein expression. The construct with a double intron had an even stronger effect, providing an extremely high level of E1 protein expression (Figure 6A, lanes 10-13).

We also measured the functional activity of the expressed E1 protein in transient replication assays. The DpnI-resistant extrachromosomal DNA replication signals of HPV11URR were analyzed by Southern blotting with an HPV11URR-specific radiolabelled probe. At a constant E2 level, the effects of different E1 expression levels from the various E1 expression plasmids were also reflected in the Southern blot replication assay, where E1 protein expressed from the L+ and L- plasmids supported URR-containing plasmid replication according to the observed E1 expression levels (Figure 6C, lanes 1-12). E1 from the plasmid containing one intron restored the effect of the L+ system (compare lanes 13-18 and lanes 1-6), and E1 from the plasmid containing two introns supported DNA replication most efficiently. The replication signal was detectable when a concentration as low as 1 ng was used (lanes 19 and 20), and clear toxicity was observed in cells at concentration of 1000 ng (lanes 25 and 26).

### Mapping of HPV11 E1 mRNA 5′ ends transcribed from various E1 expression vectors

**Figure 7:**
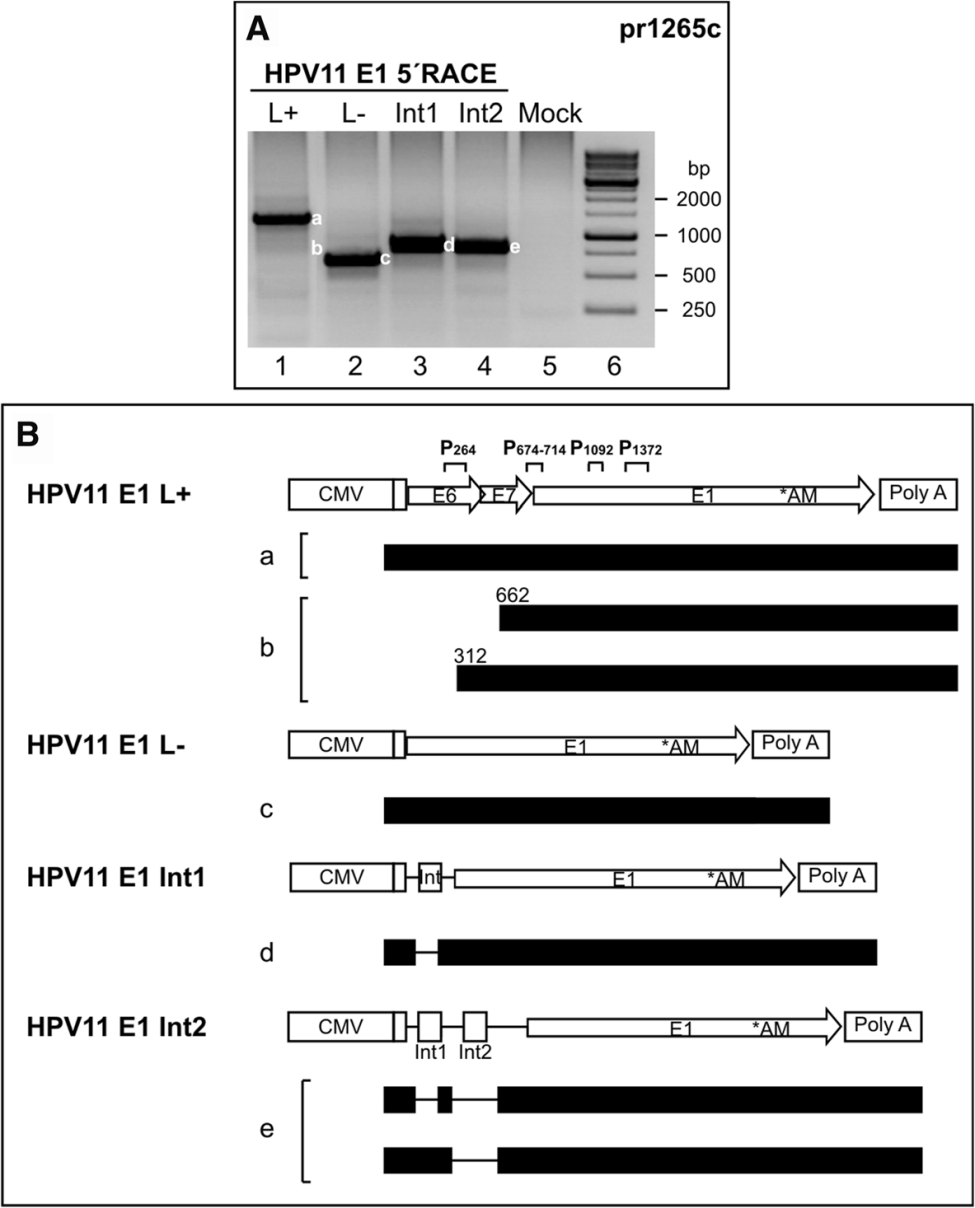
Mapping of HPV11 promoter activity from E6/E7-containing E1 expression plasmids. **(A)** U2OS cells were transfected with 1 μg of different HPV11 expression plasmids (indicated at top of panel A and schematically introduced in panel B). PolyA^+^ mRNA was extracted at 24 h post-transfection, and 5′ RACE analysis was performed with the HPV11-specific primer pr1265c. The products from a-e were sequenced, and their structures are shown in panel B. Mock-transfected U2OS cells were used as a negative control (lane 5). **(B)** Schematic map of the 5′ RACE products from the E1 expression plasmids, together with HPV11 E1 L+, L-, Int1 and Int2 expression vector maps. All of the E1 protein-coding transcripts (indicated with letters from a-e, shown at left) are shown, indicating their exons (solid boxes), introns (lines) and potential TSSs in E6 and E7 ORFs (nt numbers).

All of the previously described HPV11 promoters have been mapped to the E6/E7 protein coding region at nt 90, nt 264 and nt 674-714. In our L+ E1 expression system, the first viral promoter P90 is disrupted, and the E6 ORF starts at nt 105, whereas the remaining promoters and potential splice donor sites at leader sequence are intact in the E6/E7 ORF region. The CMV promoter is an extremely strong promoter; however, viral promoters may also function and initiate transcription on their own or splicing may still contribute to the generation of translatable E1 mRNA. Therefore, we searched for evidence of functional viral promoters in the L+ E1 expression vector or for evidence of a splicing event occurring in the E6/E7 ORF, as in the case of the high-risk HPVs. One microgram of different E1 expression vectors was transfected into U2OS cells, and polyA^+^ mRNA was extracted at 24 h post-transfection. A 5′ RACE analysis was performed with the HPV11-specific primer pr1265c. All of the observed 5′ RACE products shown in Figure 7A were gel purified, cloned and sequenced, and the results are shown in Figure 7B. As expected, strong signals could be detected from transcripts starting from the CMV promoter region. The E1 transcript from the L+ expression vector was unspliced, and the CMV promoter mimicked the early promoter at P90 (Figure 7A and B, species a). The L- mRNA was also unspliced, and the CMV promoter served as the late promoter at P674-714 (Figure 7A and B, species c). The insertion of the intron between the CMV promoter and the E1 coding region (Int1) showed that the intron was spliced out in all of the sequenced clones (Figure 7A and B, species d). The E1 expression vector Int2, which contained two introns between the CMV promoter and the E1 coding region, provided the following double-spliced products: a) both of the introns were spliced out or b) a single intron was spliced, leaving the first intron intact (75% of clones) (Figure 7A and B, species e). A weak signal could be detected for the L+ sample at the level of the 750 bp size marker (Figure 7A and B, species b). The analysis of this product showed that the viral promoters were functional in the presence of the CMV promoter (Figure 7A and B, species b) because we were able to detect the following two transcripts: one starting at P264 and the other at the P674-714 promoter region. However, we did not detect any splicing event occurring in the E6/E7 region.

We conclude that the E1 plasmids with introns between the CMV promoter and the E1 gene restored E1 expression and the functionality of E1 as a replication helicase. Therefore, different α-HPVs use different strategies for the production of the E1 replication factor. Thus, high-risk viruses generate E1-encoding mRNA via splicing, whereas low-risk viruses use their full leader sequence for expressing a biologically active E1 protein.

## Discussion

Previously, we have used the human osteosarcoma cell line U2OS to study α- and β-HPV DNA replication and gene expression patterns [13-17]. Our analyses employing the HPV11wt and E8- genomes in U2OS cells showed that the events of the papillomavirus three-step replication cycle could be mimicked, as in native keratinocyte cell lines, including the first amplification of the virus genome; the latent phase, where the genome copy number is stable; and the second genome amplification phase. The initiation of the replication of the HPV11wt genome, and particularly the E8- genome, was efficient in U2OS cells. Some differences in maintenance were observed when HPV11-positive cells were grown as actively proliferating cultures versus slow dividing dense cultures; intensive passaging of the cells led to some reduction of the viral genome copies for both, the HPV11wt and E8- genomes. The same tendency was also observed for the subconfluently cultivated low-risk HPV11 and HPV6b subclones when we introduced re-ligated HPV genomes into U2OS cells [13]. The high-risk HPV18 and HPV16 maintain stably their genomes in the cells under subconfluent conditions with active passaging [13]. Subclones of the β-papillomavirus HPV5 and HPV8 also maintain their copy numbers under subconfluent conditions [13,15]. In contrast, under confluent culture conditions, the HPV11wt genomes are effectively maintained in the U2OS cells, while in the case of HPV11E8- mutant 5 to 6 times amplification of the genome occurred. These data indicate that the amplification of the HPV11 E8- mutant genomes under confluent culture conditions generally exhibits the features of α-HPV replication, as previously described for the α-papillomaviruses HPV18 and HPV16 [13,17]. Notably, the β-papillomavirus HPV5 is not capable of amplifying the replication signal in confluent cultures of U2OS cells [15]. One of the reasons may be the difference in the structural organization of the URR regions and regulation of gene expression between the α- and β-papillomaviruses or the response to certain positive cellular factors capable of activating viral replication, which are expressed at considerably higher levels compared with those of subconfluent cultures. Alternatively, cell division in confluent cultures is slowed down, which allows the viral genome to replicate for a longer period because HPV genomes are not restricted to once-per-cell cycle replication, and genome replication occurs not only in S phase but also in G2 phase [11,14,40].

The transcriptional data obtained from the transiently transfected HPV11-positive U2OS cell line are identical to the transcripts found in condylomata acuminata [20], experimental condylomata [23] and the human squamous carcinoma cell line SCC-4 [26]. Indications of transcription events from late region genes could be observed from both promoter analyses and the mapping of the polyA signal. However, the isolation of intact mRNAs from the L1 and L2 genes was unsuccessful, indicating that these transcripts are not stable and that the L1 and L2 structural proteins are not expressed in U2OS cells. Although the second amplification of the viral genome could be observed, the expression of the late protein mRNA was not detected.

The E8 ORF is highly conserved among α-HPVs. During HPV11 transcripts analyses, we identified two mRNAs with an E8 ORF within their sequences: one possibly encoding the E8**^**E1 fusion protein and a second possibly encoding the E8**^**E2 fusion protein. The fusion protein E8**^**E2 is responsible for the repression of viral protein expression, including that of the viral replication proteins E1 and E2 [15,17,31,34]. Mutation of the E8 start codon in the HPV11 genome increase DNA replication by at least 6-fold. However, in the case of the HPV5, HPV18 and HPV31, the effect on DNA replication initiation is even greater, ranging from 30- to over 100-fold [15,17,34]. Analyzing the expression of the E8**^**E1 and E8**^**E2 fusion proteins and their effect on HPV11E8- genome replication in the present study revealed that the E8**^**E1 protein did not affect DNA replication and may be important in other aspects of the HPV11 life cycle. In contrast, the E8**^**E2 protein repressed genome replication in a concentration-dependent manner. Analyzing the HPV11E8- genome mRNA transcripts revealed that the E8**^**E2 protein negatively regulates its own mRNA transcription, in addition to repressing the transcription of E1 and E2 mRNAs, suggesting that this protein is part of the negative regulatory loop for the viral gene expression.

Analysis of the transcripts obtained from HPV11-positive U2OS cells indicated that the only transcript encoding the E1 replication protein is the polycistronic mRNA consisting of all of the early genes in the HPV11 genome: E6, E7, E1, E2, E4, E5a and E5b. Previous studies of HPV11 transcripts and E1 protein expression have suggested that E1 can also be translated from mRNA in which the TSS is at the end of the E7 ORF; therefore, the E6 and E7 genes are absent [20]. To study this difference, we designed two E1 expression vectors: one containing the E6 and E7 leader sequence and a second in which the leader sequence was absent. The E1 protein from the L+ plasmid was functional and supported DNA replication, whereas the E1 protein from the L- plasmid was not expressed and did not support viral DNA replication as effectively as that from the L+ plasmid. This difference might be explained by mRNA stability or by translational effectiveness. In the case of high-risk α-HPVs (e.g., HPV16, HPV18, and HPV31), an intron within the E6 ORF that is spliced in E1-containing mRNAs, which might be the key event in the expression of biologically active E1 protein [17,36,38,39]. To study this possibility in the HPV11 system, we generated E1 expression vectors in which the leader sequence was substituted with one or two heterologous artificial introns. The splicing event between the promoter and E1 gene restored the effect of the leader sequence. The E1 protein was expressed effectively and supported viral DNA replication. Two introns have an even greater effect on E1 protein expression and activity. In contrast, the analysis of the transcripts obtained from the L+ sequence did not reveal any introns within the leader sequence, indicating that other regulatory factors in the HPV11 E6/E7 region assure effective and biologically active E1 protein expression.

## Conclusions

The analysis of HPV11 replication and mapping of the mRNA transcripts indicated that U2OS cells provide an adequate environment for HPV11 replication and gene expression. Considering the immortalized state and ease of cultivation of this cell line, this system could be a valuable tool for papillomavirus molecular biology-related studies, as well as for the development of high-throughput analyses for the screening and validation of anti-HPV drugs.

## Materials and methods

### Cell lines and transfection

U2OS cells obtained from the American Type Culture Collection (ATCC) (number HTB-96) were grown in Iscove’s modified Dulbecco’s medium (IMDM) supplemented with 10% fetal calf serum (FCS). The cells were transfected via electroporation (220 V, 975 μF) using a Bio-Rad Gene Pulser II apparatus supplied with a capacitance extender (Bio-Rad Laboratories). Episomal HPV11 genome amplification was achieved by growing transfected cells under confluent conditions for 10 days without passaging. At 48 h post-transfection, the cells were subjected to geneticin sulfate G418 selection (final concentration, 400 μg/ml, BioTop) to eliminate untransfected cells. The medium was changed every second day. Total DNA was extracted at the time points indicated below.

### Plasmids

The HPV11 genome sequence and its numbering are based on the sequence of GenBank accession no. M14119. Minicircle producing plasmid backbone pMC.BESBX [41] was inserted into BamHI site in the HPV11 genome L1 region (between nt 7072 and 7073 in HPV11 genome). Almost all of bacterial backbone is removed during minicircle plasmid preparation [14]. The final product is circular HPV11 genome DNA with 91 bacterial backbone derived nucleotides between positions 7072 and 7073. For the HPV11E8- mutant genome, the ATG start codon from pMC.BESPXHPV11wt (at the nt 1242 position in HPV11 genome) was mutated to ACG. To produce HPV11 genome for electroporation: HPV11wt and HPV11E8- minicircle genomes were produced from the parental plasmids in *E. coli* and purified as described by Reinson et al. [14]. To achieve complete removal of the unrecombined parental plasmids and the pMC.BESPX vector, the plasmids extracted from the bacterial cells were subjected to an additional gel purification step.

The E8^E1 expression vector pQMCFH11E8E1 was generated by amplification of the HPV11 E1 C-terminus from the plasmid pMC.BESPXHPV11wt with the primers 11E8-E1_F_NheI (5′-TCTGCTAGCGCCACCATGGCTATTCTGAAGTGGAAGCTGCAACGCAGAAATGGGAATGCAGTATATGAACTATC-3′) and 11E1_R_Bsp (5′-CCCTTCGAATCATAAAGTTCTAACAACTGATCCTG-3′). The E8^E2 expression vector pQMCFH11E8E2 was generated by amplification of the HPV11 E2 C-terminus from the pMC.BESPXHPV11wt plasmid with the primers 11E8-E2_F_NheI (5′-TCTGCTAGCGCCACCATGGCTATTCTGAAGTGGAAGCTGCAACGCAGCACTGTACGAGAAGTATCCATTG-3′) and 11E2_R_Bsp119I (5′-CCCTTCGAATTACAATAAATGTAATGACATAAACCC-3′). The PCR products were cleaved with the enzymes NheI and Bsp119I (Thermo Scientific) and inserted into the vector pQMCF_MCS_9 (Icosagen Cell Factory Ltd.), which was opened through NheI/Bsp119I digestion.

The cloning of the HPV11 URR plasmid and the L+ E1 and E2 expression vectors was described previously by Kadaja et al. (designated pUCURR-11, pMHE1-11 and pQMNE2-11, respectively) [37]. The HPV11 L- E1 expression vector was obtained by amplification of the E1 ORF from the HPV11 L+ E1 expression vector (nt 823 to 2781 in the HPV11 genome) using the primers E1.Xba-11 (5′-CCTCTAGATCCATGGCGGACGATTCA-3′) and E1.3.11 (5′-GGAAGCTTTTCTTCATAAAGTTCTAACAACTGATCC-3′). The PCR product was cleaved with XbaI and HindIII and inserted into the XbaI/HindIII site of the eukaryotic expression vector pQM-Ntag/Ai + (Icosagen Cell Factory Ltd.). The HPV11 Int1 and Int2 E1 expression vectors were obtained by cleaving the vector pRL-TK (Promega) with the enzymes HindIII and NheI and by cloning the pRL-TK intron-containing region into the XbaI site of the HPV11 L- vector. By blunt-end cloning, we obtained E1 expression plasmids with one (Int1) or two (Int2) introns between the CMV promoter and the E1 gene. All of the modifications of the HPV11 L+ E1 expression vector described in Kadaja et al. were also performed for the L-, Int1 and Int2 E1 expression plasmids [37].

### RNA extraction and rapid amplification of cDNA ends (RACE)

U2OS cells were transfected with 500 ng of the HPV11wt or HPV11E8- genome, and samples used for establish the stable expression were transfected with 2 μg of the linearized pBabe-Neo construct. PolyA^+^ RNA templates were extracted from cells using a Micro-FastTrack™ 2.0 Kit (Invitrogen) at 4 and 10 days post-transfection. Then, 275 ng or 350 ng of polyA^+^ RNA was used as a template for 5′ or 3′ RACE analysis, respectively. Both the 5′ and 3’ RACE assays were performed using a SMARTer™ RACE cDNA Amplification Kit (Clontech) according to the manufacturer’s instructions. The positions and the sequences of the HPV11-specific primers used for the amplification of the RACE products are shown in Figure 2. The amplified RACE products were purified from agarose gels, cloned and fully sequenced as single clones.

### RT-PCR

Samples of 200 ng of polyA^+^ RNA extracted from cells using a Micro-FastTrack™ 2.0 kit (Invitrogen) were subjected to DNase treatment with a Turbo DNA-free Kit (Ambion), and cDNA was synthesized using a Superscript III First Strand cDNA synthesis Kit (Invitrogen) according to the manufacturer’s instructions. Then, 4 μl of cDNA was used in a single PCR reaction, along with 4 μl of 5× HOT FIREPol® Blend Master Mix with BSA, 12.5 mM MgCl_2_ (Solis Biodyne) and the HPV11-specific primers pr1241 and pr3692c (Figure 2) in a 20 μl of total reaction volume. Amplification of the newly synthesized cDNA was performed using the following program: 95°C 15′, 94°C 30″, 65°C 30″, and 72°C 2′, for a maximum of 25 cycles. The RT-PCR products were purified from agarose gels, cloned and fully sequenced as single clones.

### Replication analysis

Extrachromosomal DNA was extracted from the transfected cells at 1 to 5 days post-transfection via the modified Hirt method [42], or total genomic DNA was extracted at 10 to 21 days post-transfection from U2OS cells as described previously [13]. Before Southern blot analysis, half of each extrachromosomal DNA sample or 3 μg of each total DNA sample was treated with the linearizing enzyme HindIII, and non-replicated, bacterially produced dam-methylated input DNA was fragmented with DpnI (Thermo Scientific). The DNA samples were resolved on a 0.8% (extrachromosomal DNA) or 0.6% (total genomic DNA) agarose gel using 1× Tris-acetate-EDTA (TAE) buffer. Electrophoresis was performed at 0.8 V/cm for 20 h. The separated DNA fragments were treated with 0.1 M hydrochloric acid, denatured with Sol A (0.5 M NaOH, 1.5 M NaCl), neutralized with Sol B (1 M Tris pH 7.4, 1.5 M NaCl), transferred to a Hybond-N+ membrane (Amersham Pharmacia Biotech) via the Southern blot method and hybridized with a radiolabelled HPV11-specific probe. HPV11 replication signals were detected via exposure to X-ray film.

### Western blotting

Cells in a 60 mm culture dish were washed twice with 1× PBS, detached from plates with PBS-3 mM EDTA (pH 7.5) and collected via centrifugation. The cells were lysed with 1× Laemmli buffer (4% SDS, 20% glycerol, 120 mM Tris-Cl (pH 6.8), and 200 mM DTT), boiled at 100°C for 5 min and centrifuged for 10 min at 14000×g. The proteins were separated in 8 to 10% polyacrylamide-SDS gels and transferred to Immobilon-P membranes (Millipore, USA). The HPV11 E1 protein with a hemagglutinin (HA) epitope tag was detected using the rat polyclonal antibody 3F10-HRP (12013819001; Roche), which was diluted 1:500. An antibody against the cellular marker α-tubulin (B-512, Sigma-Aldrich) was used at a dilution of 1:5000. For visualization, a secondary α-mouse-HRP antibody (LabAS) was used at a dilution of 1:10000.
